# Toward Development of a Diabetic Synovium Culture Model

**DOI:** 10.3389/fbioe.2022.825046

**Published:** 2022-02-21

**Authors:** Neeraj Sakhrani, Andy J. Lee, Lance A. Murphy, Hagar M. Kenawy, Christopher J. Visco, Gerard A. Ateshian, Roshan P. Shah, Clark T. Hung

**Affiliations:** ^1^ Department of Biomedical Engineering, Columbia University, New York, NY, United States; ^2^ Department of Rehabilitation and Regenerative Medicine, Columbia University, New York, NY, United States; ^3^ Department of Mechanical Engineering, Columbia University, New York, NY, United States; ^4^ Department of Orthopedic Surgery, Columbia University, New York, NY, United States

**Keywords:** synovium, type 2 diabetes, insulin resistance, hyperglycemia, osteoarthritis, calcium signaling, shear stress, primary cilia

## Abstract

Osteoarthritis (OA) is a degenerative joint disease characterized by articular cartilage degradation and inflammation of synovium, the specialized connective tissue that envelops the diarthrodial joint. Type 2 diabetes mellitus (DM) is often found in OA patients, with nearly double the incidence of arthritis reported in patients with diabetes (52%) than those without it (27%). The correlation between OA and DM has been attributed to similar risk factors, namely increasing age and joint loading due to obesity. However, a potential causative link is not well understood due to comorbidities involved with treating diabetic patients, such as high infection rates and poor healing response caused by hyperglycemia and insulin resistance. The purpose of this study was to investigate the effect of hyperglycemic and insulin culture conditions on synovium properties. It was hypothesized that modeling hyperglycemia-induced insulin resistance in synovium would provide novel insights of OA pathogenesis in DM patients. To simulate DM in the synovial joint, healthy synovium was preconditioned in either euglycemic (EG) or hyperglycemic (HG) glucose concentrations with insulin in order to induce the biological response of the diseased phenotype. Synovium biochemical composition was evaluated to determine ECM remodeling under hyperglycemic culture conditions. Concurrent changes in AKT phosphorylation, a signaling pathway implicated in insulin resistance, were measured along with gene expression data for insulin receptors, glucose transporters, and specific glycolysis markers involved in glucose regulation. Since fluid shear stress arising during joint articulation is a relevant upstream stimulus for fibroblast-like synoviocytes (FLS), the predominant cell type in synovium, FLS mechanotransduction was evaluated via intracellular calcium ([Ca^2+^]_i_). Incidence and length of primary cilia, a critical effector of cell mechanosensing, were measured as potential mechanisms to support differences in [Ca^2+^]_i_ responses. Hyperglycemic culture conditions decreased collagen and GAG content compared to EG groups, while insulin recovered ECM constituents. FLS mechanosensitivity was significantly greater in EG and insulin conditions compared to HG and non-insulin treated groups. Hyperglycemic treatment led to decreased incidence and length of primary cilia and decreased AKT phosphorylation, providing possible links to the mechanosensing response and suggesting a potential correlation between glycemic culture conditions, diabetic insulin resistance, and OA development.

## Introduction

Osteoarthritis (OA), the most common musculoskeletal disorder, is a degenerative condition that affects an estimated 250 million people worldwide (Rosa et al., 2009; Hamada et al., 2016; Stefani et al., 2019). OA is characterized by articular cartilage degradation, altered subchondral and peri-cartilaginous bone, and synovial inflammation (King and Rosenthal, 2015; Silverstein, 2017). The prevalence of comorbidities among patients with OA is high, contributing to gastrointestinal, neurological, cardiovascular, and endocrine complications (Swain et al., 2020). Type 2 diabetes mellitus (DM) is a common comorbidity in OA patients, contributing to high infection rates and poor healing (King and Rosenthal, 2015; Swain et al., 2020). DM is characterized by chronic hyperglycemia caused by a combination of insulin resistance and a deficiency of insulin secretion or action (Hamada et al., 2016; Röder et al., 2016; Galicia-Garcia et al., 2020).

The association between OA and DM is well supported. More than 90% of patients diagnosed with DM are obese (Bramante et al., 2017). Joint loading due to obesity has been linked to articular cartilage damage in the development and progression of OA (Bramante et al., 2017; Silverstein et al., 2017; Sun et al., 2021). Increasing age is another known cause for the development of both diseases as adults above the age of 65 are more than twice as likely to develop either condition (Piva et al., 2015; Veronese et al., 2019). While the separate incidences of both OA and DM are significant, overlap between the two diseases are also common. DM is often found in OA patients, with higher occurrence of arthritis reported in patients with diabetes (52%) than those without it (27%) (Piva et al., 2015). Conversely, patients diagnosed with OA have a 61% greater risk of developing DM compared to people without arthritis (Dong et al., 2017).

While the connection between OA and DM has historically been attributed to similar risk factors including increasing age and joint loading due to obesity, the effects of hyperglycemia and insulin resistance on synovium and their contribution to OA pathogenesis have not been thoroughly investigated due to the associated comorbidity involved with treating this patient population (Hamada et al., 2016). The synovium is a specialized connective tissue that envelops arthrodial joints and is comprised mainly of fibroblast-like synoviocytes (FLS) (Estell et al., 2017). The tissue maintains the synovial fluid-filled region that delivers a lubricating environment for the articulating cartilage surfaces and serves as a semi-permeable membrane that facilities solute transport across the joint (Blewis et al., 2010; Tamer, 2013; Estell et al., 2017). To this end, this research, comprised of four complementary studies, investigated the effect of hyperglycemic treatment and insulin culture conditions on synovium properties toward the development of an *in vitro* diabetic synovium model for basic science and translational research applications.

Insulin production and release has been shown to play a protective role in regulating synovial inflammation and catabolism in patients with DM (Griffin and Huffman, 2016; Song et al., 2021). While limited studies have directly investigated the role of insulin on synovium, the anabolic effects of insulin have been thoroughly investigated in a variety of other musculoskeletal tissues including bone, cartilage, and tendon where insulin serves as a vehicle that stimulates cell differentiation, proliferation, and extracellular matrix (ECM) production (Weiss and Reddi, 1980; Maor et al., 1993; Griffin and Huffman, 2016; Wu et al., 2017; Frost et al., 2018; Durgam et al., 2019; Cipriani et al., 2020). However, insulin signaling and metabolic function are impaired under diabetic conditions. This decrease in the protective role of insulin contributes to high infection rates, joint inflammation, and a poor healing response, characteristic of the arthritic disease state (Bustamante et al., 2017). Since glucose and insulin treatments have been implicated in ECM remodeling, study 1 investigated the effect of hyperglycemic and insulin culture conditions on tissue biochemical composition by looking at DNA, glycosaminoglycan (GAG), and collagen content of healthy and OA human synovium explants (Williams et al., 2015). Histological characterization of GAG and collagen distribution was assessed along with expression of glucose transporter 1 (GLUT1), as a specific marker of glucose transport in explants exposed to hyperglycemic culture conditions (Rosa et al., 2009; Li et al., 2021).

**FIGURE 1:**
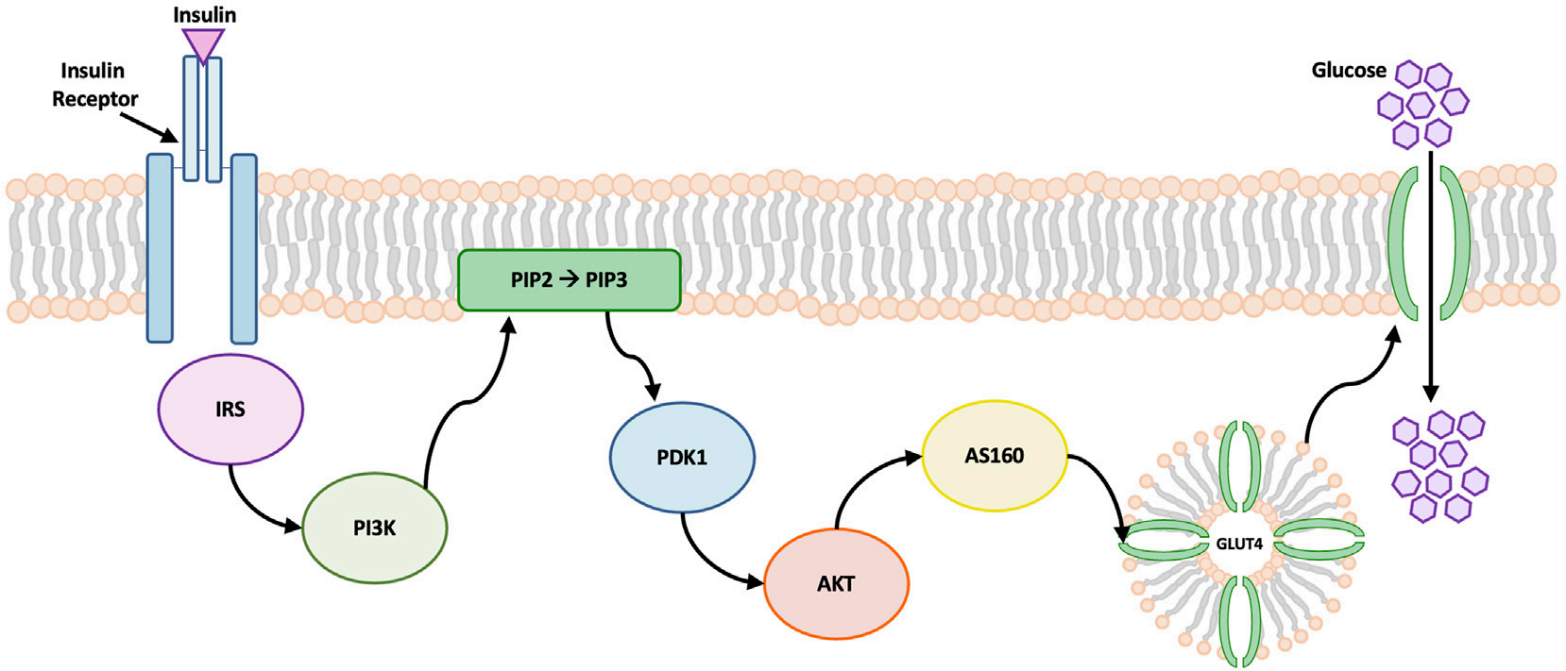
Schematic of AKT signaling pathway. Insulin binding to cell surface receptors initiates the signal transduction cascade. Insulin promotes the phosphorylation of AKT which leads to glucose transporter 4 (GLUT4) translocation to the cell membrane. GLUT4 contains specialized vesicles that permit fusion with the cell membrane, allowing glucose uptake into the cell (Wang et al., 2020). Abbreviations: insulin receptor substrates (IRS), phosphatidylinositol 3-kinase (PI3K), 3-phosphoinositide-dependent kinase 1 (PDK1), phosphatidylinositol-4, 5-bisphosphate (PIP2), phosphatidylinositol-3, 4, 5-triphosphate (PIP3).

Patients with DM can suffer compromised synovium function, as the development of synovial insulin resistance under hyperglycemic conditions reduces the ability of insulin to regulate glucose levels and suppress the production of inflammatory mediators known to stimulate the progression of OA (King and Rosenthal, 2015; Griffin and Huffman, 2016). In DM, the AKT signaling pathway is involved in the regulation of insulin and glucose uptake in adipose tissue (Hamada et al., 2016; Hatting et al., 2018). The AKT signaling cascade begins with the binding of insulin to surface protein receptors. Insulin stimulates the phosphorylation of AKT, causing the translocation of glucose transporters (GLUT) to the cell membrane, which facilitates glucose uptake into the cell (Figure 1) (Mackenzie and Elliott, 2014; Tchetina et al., 2020). Toward efforts to better understand markers of insulin resistance or compromised insulin response in synovium exposed to hyperglycemic and insulin culture conditions, study 2 characterized concurrent changes in AKT phosphorylation and specific gene expression markers implicated in glucose activity and insulin resistance for healthy and non-diabetic OA synovial explants.

While the AKT pathway is a downstream marker of DM and OA, it is regulated by upstream calcium influxes (Danciu et al., 2003; Nicholson-Fish et al., 2016). Calcium, a ubiquitous second messenger, is one of the earliest cell signaling events activated by applied physical and chemical stimuli (Yellowley et al., 1999; Estell et al., 2017). In diabetic patients, insulin resistance contributes to a decreased cellular response to external stimuli (Wilcox, 2005; Petersen and Shulman., 2018). However, synoviocytes respond to these mechanical cues to support joint health and modulate inflammation (D’Andrea et al., 1998; Sun et al., 2003; Yanagida-Suekawa et al., 2013, Estell et al., 2017; Falconer et al., 2021). Study 3 monitored intracellular calcium response ([Ca^2+^]_i_) to fluid-induced shear stress of healthy and non-diabetic OA FLS that are cultured in hyperglycemic and insulin conditions.

To further explore potential mechanisms for intracellular calcium response to shear stress between the hyperglycemic and insulin treated FLS groups, the effect of glucose on the incidence and length of primary cilia was investigated. Primary cilia are non-motile, microtubule-based organelles that emanate from the cell surface in a variety of vertebrate cells including synoviocytes, and are essential structures implicated in cell mechanotrandsuction (Malone et al., 2007; Lu et al., 2008; Ou et al., 2009; Besschetnova et al., 2010; Rattner et al., 2010; Hoey et al., 2012; Estell et al., 2017; Wheway et al., 2018). A decrease in the incidence of primary cilia has been shown to cause less mechanotransduction, contributing to decreased percentage of cells that respond to fluid shear (Estell et al., 2017). Furthermore, increased glucose concentrations lower the number of ciliated cells (Gerdes et al., 2014; Takahashi et al., 2018). Therefore, study 3 also characterized the incidence and length of primary cilia for healthy and OA FLS in order to determine the effect of hyperglycemia and insulin exposure on cilia properties, providing a potential link between FLS mechanosensitivity and fluid shear.

For healthy and non-diabetic OA synovium, upstream markers of cellular activity *via* intracellular calcium response and primary cilia properties were linked with downstream markers of compromised glucose activity and insulin resistance via characterization of AKT phosphorylation under hyperglycemic culture conditions. This model of preconditioning with hyperglycemic and insulin treated media can be optimized to confer the diabetic phenotype. Study 4 investigated the effect of hyperglycemic culture conditions and insulin exposure on AKT phosphorylation and upstream markers of cellular activity using diabetic OA synovium. We anticipate that the results from explants containing both comorbidities can be used to further develop an *in vitro* model of diabetic insulin resistance. By optimizing culture conditions, the diseased phenotype can be conferred from healthy tissue in order to overcome variability associated with age or disease state of patients undergoing total knee arthroplasty.

## Materials and Methods

### Synovium Explant Harvest and Preparation

Human OA synovium explants were harvested (IRB #AAAQ2703) from the region of the joint capsule adjacent to the medial and lateral femoral condyle of three diabetic and non-diabetic subjects each (age 72 ± 7 and 65 ± 6 years old, respectively) during total knee arthroplasty (Table 1) (Stefani et al., 2019). Human synovium from three healthy donors (age 21 ± 2 years old) was obtained from the Musculoskeletal Transplant Foundation (Edison, NJ). Explants were trimmed of excess adipose and outer capsule tissue prior to digestion and cell isolation (Stefani et al., 2019). Human synovium samples were kept separate by donor for experiments.

**TABLE 1:** Human donor information. Phenotypes: **(A–C)** diabetic OA, **(D–F)** non-diabetic OA, **(G–I)** healthy. Bold values denote average age and standard deviation of each donor phenotype; Diabetic OA Human (Mean ± SD) denote donors A–C. OA Human (Mean ± SD) denote donors D–F. Healthy Human (Mean ± SD) denote donors G–I.

Donor ID	Gender	OA grade	Age	Diabetes
A	M	4	80	✓
B	F	4	69	✓
C	F	4	66	✓
D	F	4	60	✕
E	F	4	63	✕
F	F	4	71	✕
G	M	-	19	✕
H	M	-	21	✕
I	M	-	22	✕
Diabetic OA Human (Mean ± SD)			**72 ± 7**	
OA Human (Mean ± SD)			**65 ± 6**	
Healthy Human (Mean ± SD)			**21 ± 2**	

Synovial explants were individually cultured for 7 days in varying glucose and insulin treated groups (*n* = 3 donors per group, 2 explants/donor). Explants were treated in serum-free Dulbecco’s Modified Eagle’s Medium (DMEM; Gibco) and separated into two groups supplemented with different glucose concentrations: euglycemic (EG; 5.56 mM D-glucose) and hyperglycemic (HG; 12.5 mM D-glucose). Physiologic levels of human recombinant insulin (6.25 μg/ml) were added to both EG and HG groups (EGI and HGI, respectively). Individual explant specimens were cultured in 5 ml media, with media changes three times per week. Synovium explants were collected at day 0 (initial harvest, start of treatment) and day 7 (end of glycemic, insulin treatment).

### Biochemistry

Human healthy and OA explants were frozen at −20°C and lyophilized overnight. Samples were weighed and solubilized by incubating for 16 h at 56°C in 0.5 mg/ml Proteinase K (Cat. No. 193504; MP Biomedicals) and Proteinase K buffer containing 50 mM Tris saline, 1 mM EDTA, 1 mM iodoacetamide (Cat. No. 12227-1000; Acros), and 10 mg/ml Pepstatin A (Cat. No. BP2671100; Fisher) (Riesle et al., 1998). DNA content was analyzed using Picogreen (Cat. No. P11496; ThermoFisher), while collagen content was analyzed using an orthohydroxyproline (OHP) assay with a 1:7.64 OHP-to-collagen mass ratio (Stegemann and Stalder, 1967). GAG content was analyzed using the 1,9-dimethylmethylene blue dye-binding assay (Product No. 341088; Sigma-Aldrich).

### Media Analysis

Media samples from the synovial explants treated with glucose and insulin were collected during each media change. Day 7 media samples were assayed using Griess Reagent Kit for nitric oxide (NO) determination (Cat. No. G7921; ThermoFisher). Media samples were also assayed for GAG content. To account for temporal variations in the biochemical properties of the specimens, NO and GAG values were normalized to day 0 values.

### Histological Characterization

Synovium samples were fixed in 4% paraformaldehyde (PFA), embedded in paraffin wax, and sectioned into 8 µm slices. Sections were stained with hematoxylin and eosin (H&E) to determine cell and matrix distribution, Safranin O for GAG content, and Picrosirius Red to determine collagen distribution. Samples were immunohistochemically stained for GLUT1 (1:250; rabbit monoclonal; Cat. No. ab115730; Abcam) using a 3,3′Diaminobenzidine (DAB) rabbit substrate kit (Cat. No. ab64261; Abcam) following heat-mediated epitope retrieval for 10 min at 95°C in citrate buffer (pH 6.0). The sections were counterstained with Mayer’s hematoxylin (Cat. No. ab220365, Abcam).

### AKT Phosphorylation

Following the glucose (EG, HG) and insulin preconditioning period, explants were weighed and placed in lysis buffer containing protease and phosphatase inhibitor cocktail (Product No. PPC1010; Sigma-Aldrich). The tissue was homogenized via bead beating using polypropylene tubes pre-filled with 1.4 mm ceramic beads (Product No. 19-627D; Omni). Relative levels of phospho- and pan-AKT were determined for each glycemic and insulin treated group using an ELISA (Cat. No. PEL-AKT-S473-T; RayBiotech) after day 7 treatment according to the manufacturer’s instructions.

### qPCR Preparation

FLS were isolated from healthy and OA synovial explants via type II collagenase (Cat. No. NC9522060; Fisher) digestion and expanded in αMEM containing 10% fetal bovine serum (FBS), 1% PSAM and 5 ng/ml fibroblast growth factor-2 (FGF2) (Cat. No. PHG0021; Fisher) for two passages to obtain a pure population of FLS. For gene expression analysis, healthy and OA FLS were preconditioned in EG and HG medias for 7 days. Cells were collected on days 1, 3, and 7, respectively, and resuspended in RNAlater (Cat. No. AM7020; ThermoFisher). RNA precipitation was performed using Qiagen miRNeasy columns (Cat. No. 74106; QIAGEN). cDNA was synthesized from RNA using the iScript cDNA Synthesis Kit (Product No. 1708891; Bio-Rad) and diluted to 1 ng/µl concentration for PCR. Gene specific master mixes were prepared using the designed primers, and iTaq Universal SYBR Green Supermix (Product No. 1725122; Bio-Rad). RT-qPCR was run on a QuantStudio™ six Flex Real-Time PCR System (Applied Biosystems™). The following primers (Integrated DNA Technologies IDT) were chosen: glucose transporter type 1 (GLUT1), glucose transporter type 4 (GLUT4), hexokinase II (HK2), glucose-6-phosphatase (G6PD), insulin-like growth factor 1 receptor (IGF1R), insulin receptor (INSR), and AKT Serine/Threonine Kinase 1 (AKT1) (Table 2). GAPDH was used as the housekeeping gene, and all samples were normalized to EG controls for each respective timepoint using the 2^−ΔΔCt^ method.

**TABLE 2:** qPCR Primer Sequences for glucose metabolism genes. GLUT1: Glucose transporter type 1, GLUT4: Glucose transporter type 4, HK2: Hexokinase, G6PD: glucose-6-phosphate-dehydrogenase, IGF1R: Insulin-like growth factor 1 receptor, INSR: Insulin receptor, AKT1: AKT Serine/Threonine Kinase 1, GAPDH (housekeeping gene).

Gene	FWD primer sequence	REV primer sequence
GAPDH	CAA GAG CAC AAG AGG AAG AGA G	CTA CAT GGC AAC TGT GAG GAG
GLUT1	GGT CAG GCT CCA TTA GGA TTT	CCC AAC TGG TCT CAG GTA AAG
GLUT4	CCA GGA TCG GTT CTT TCA TCT T	CAT CTT CGG AGC CTA TCT GTT G
HK2	TGT GAG GTC CAC TCC AGA T	GAG CCC ATT GTC CGT TAC TT
G6PD	TAG GCA GCC TCT CTG CTA TAA	TGG GCT GTT TGC GGA TTT A
IGF1R	TTC TCC CTT TCT CTC TCC TCT C	GAC AGC CAC TTC CTC AAA CT
INSR	CAT GCG GAG TTG ATG CTT TG	GCA CAG TCT CCC AGT CAA TAA
AKT1	CTA CAA CCA GGA CCA TGA GAA G	TCT TGA GCA GCC CTG AAA G

### Fluid Shear and Calcium Imaging

FLS were plated in silicone isolators (Grace Bio-Labs) on 5 μg/cm^2^ collagen type 1 coated glass slides at a cell density of 5.3 × 10^4^ cells/cm^2^ to obtain a semi-confluent layer (Estell et al., 2017). Cells were pre-conditioned in the same glucose and insulin treated groups for an additional 24 h prior to imaging, with parallel untreated controls. An osmotic control was included, consisting of EG media supplemented with sucrose (EGS).

**FIGURE 2:**
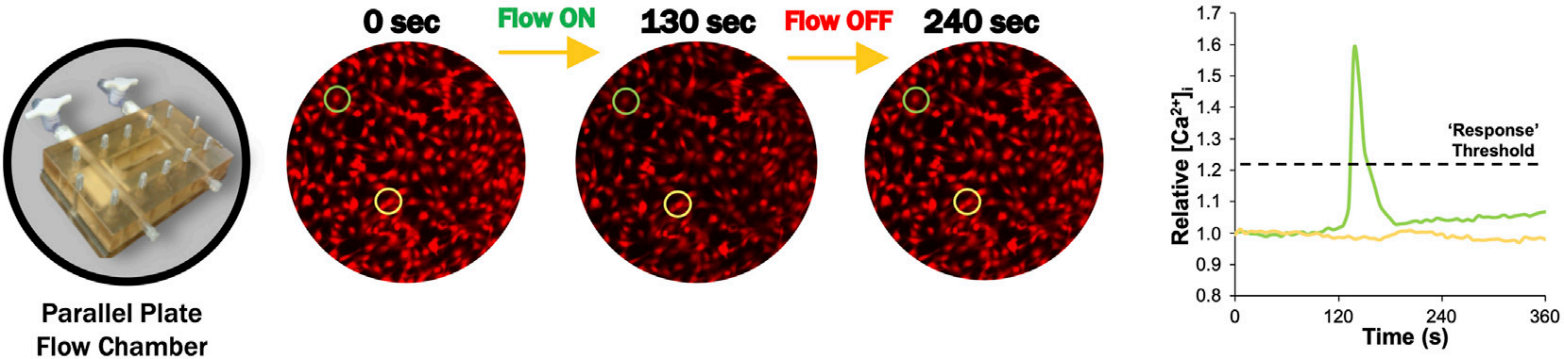
Representative FLS calcium transient to fluid-induced shear stress (Estell et al., 2017). Prior to flow (0 s), intracellular calcium response is at equilibrium. [Ca^2+^]_i_ increased after flow was initiated to a maximum value at 130 s after slight delay (Estell et al., 2017). [Ca^2+^]_i_ returned to starting equilibrium values when flow is turned off after 240 s.

Changes in intracellular calcium ([Ca^2+^]_i_) were tracked via Fura Red-AM (Cat. No. F3020; Life Technologies). FLS were stained using 5 µM Fura Red and incubated for 40 min at 37°C (Estell et al., 2017). Fluid flow-induced shear stress was applied in a parallel plate flow chamber at 0.1 Pa fluid shear stress (Figure 2). Chambers were secured on the microscope stage at room temperature, and unidirectional flow experiments were performed using Hank’s Buffered Salt Solution (HBSS) supplemented with 0.1% fetal bovine serum. Flow studies were composed of a 6-min time lapse with the following stages: a 2-min “baseline” to observe the cells at equilibrium, followed by a “stimulation” with continuous unidirectional flow at 0.1 Pa fluid shear, then 2 min of post-flow relaxation to starting baseline levels (Figure 2). Images of the time lapse were acquired at a rate of 3 s per frame.

All fluid shear data were collected for 100 cells per slide, pooled across six slides per group. Fluorescence intensity was tracked for individual cells by collecting intensity versus time measurements for a circular region within the cell body, where increasing [Ca^2+^]_i_ resulted in decreased fluorescence (Estell et al., 2017). Fluorescence intensity was normalized to the average intensity during baseline and inverted to represent relative [Ca^2+^]_i_ (Estell et al., 2017). A custom Matlab (Mathworks) code was developed to calculate the percent of responding cells. Cells were considered responders if relative [Ca^2+^]_i_ increased 20% above baseline equilibrium measurements, which was found to be sufficient to exclude any false responses due to natural variations in equilibrium levels (Figure 2) (Estell et al., 2017).

### Primary Cilia Incidence and Length Measurements

FLS were preconditioned in glycemic and insulin treated groups (EG, EGI, HG, HGI) for 24 h prior to fixation in 4% PFA and immunocytochemical staining with 2 mg/ml Alexa-488-tagged alpha-acetylated tubulin (Cat. No. sc-23950; Santa Cruz Biotechnology) for primary cilia visualization (Estell et al., 2017). FLS were counterstained with DAPI (Cat. No. 62248; Life Technologies) for nuclear visualization. Primary cilia were counted using Zen Blue software (Zeiss), and cilia lengths were measured using ImageJ (National Institutes of Health).

### Statistical Analysis

GraphPad Prism version 9.2.0 (for Windows, GraphPad Software, San Diego, California, United States) was used for statistical analysis. Healthy, diabetic, and non-diabetic OA donor groups were analyzed separately using two-way ANOVA (α = 0.05) with Tukey’s post-hoc test to correct for multiple comparisons and determine significant differences between glucose and insulin treatment on biochemical composition, AKT phosphorylation, percent responding cells, and primary cilia measurements. Non-parametric data (e.g., gene expression) were analyzed via Fisher’s Exact Test with Holm-Sidak correction for multiple comparisons. For all hyperglycemic and insulin treated media groups, replicates were combined to generate an average value across all phenotypes, glycemic conditions, and insulin treatment. Statistical significance was determined at *p* < .05.

## Results

### Study 1. Biochemical Composition of Healthy and Non-Diabetic OA Synovium Under Hyperglycemic and Insulin Culture Conditions

**FIGURE 3:**
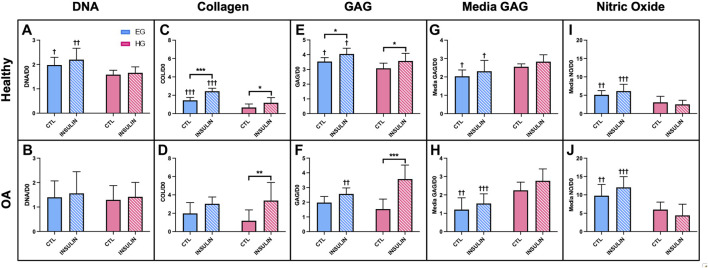
Normalized **(A,B)** DNA, **(C,D)** collagen, **(E,F)** GAG to respective day 0 value for healthy and non-diabetic OA synovium. Release of **(G,H)** GAG and **(I,J)** NO in media from glucose and insulin treated human synovium explants. †*p* < .05, ††*p* < .01, †††*p* < .0001 (EG vs. HG). *p < .05, **p < .01, ***p < .0001 (control vs. insulin).

In healthy synovium explants, HG treatment decreased DNA levels compared to EG groups for both control and insulin treated conditions (*p* = .0215 and *p* = .0009, respectively) (Figure 3A). No significant changes in DNA content were observed in either glucose or insulin treated OA specimens (Figure 3B). Healthy synovium explants exhibited decreased collagen content between EG and HG controls as well as between the parallel insulin treated groups (Figure 3C; *p* < .0001). No significant changes in collagen content were observed across both glycemic and insulin treated groups in OA synovium (Figure 3D). Comparing media groups separately, insulin treatment significantly increased collagen levels in EGI groups compared to EG in healthy explants (*p* < .0001). Compared to HG groups, HGI treatment significantly increased levels of collagen across both healthy and OA phenotypes (*p* = .0162 and *p* = .0012, respectively). HG conditions exhibited decreased GAG content compared to EG groups in healthy synovium under both control and insulin treatment (*p* = .0296 and *p* = .0242, respectively) (Figure 3E). In OA explants, insulin treatment increased GAG levels in HGI treated groups compared to EGI (*p* = .0025), but no significant changes were observed between the non-insulin treated groups (Figure 3F). Insulin treatment also significantly increased GAG content for HGI groups compared to HG controls for both healthy and diseased phenotypes (*p* = .0156 and *p* < .0001, respectively). In the healthy synovium, EGI groups also exhibited increased GAG content compared to non-insulin EG treatment (*p* = .0126).

In healthy synovium, HG treatment led to increased media GAG compared to EG for both control and insulin groups (Figure 3G; *p* = .0135 and *p* = .0129, respectively). The increase in media GAG for the HG and HGI groups were more profound in the OA explants compared to healthy synovium (Figure 3H; *p* = .0003 for control and *p* < .0001 for insulin). HG treatment decreased the release of NO in media compared to the control EG group in both the healthy and non-diabetic OA cells (Figures 3I,J; *p* = .0073 and *p* = .0092, respectively). With insulin exposure, explants also exhibited decreased NO release for HGI groups compared to EGI across both phenotypes (*p* < .0001). Comparing between phenotypes, NO release from OA explants was significantly higher (∼48% increase across all four media conditions) compared to healthy synovium.

**FIGURE 4:**
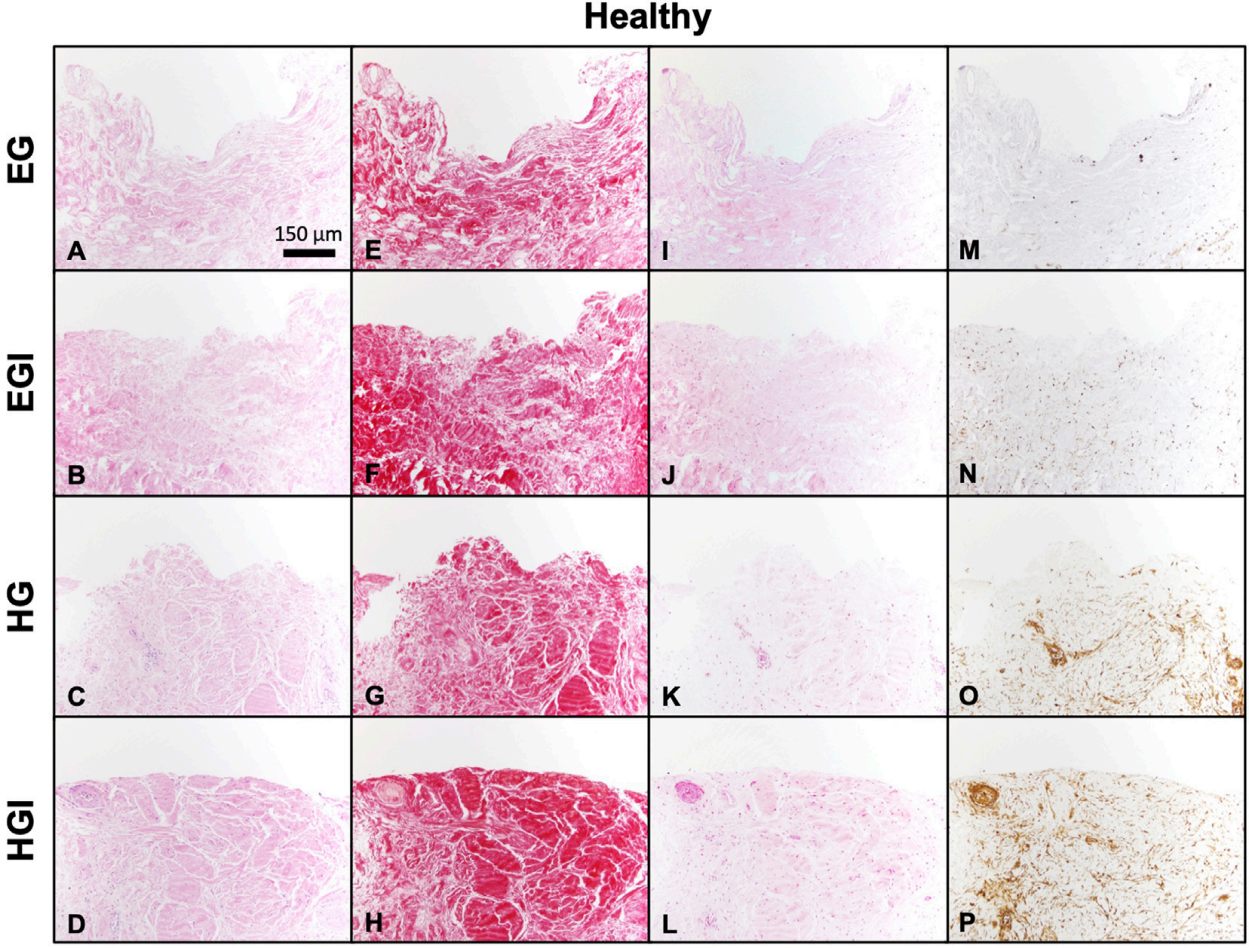
Histology of glucose and insulin treated healthy synovium explants. **(A–D)** H&E, **(E–H)** Picrosirius Red, **(I–L)** Safranin O, and **(M–P)** GLUT1.

Histological staining of healthy synovium yielded comparable results. Similarities in synovial structural morphology under hyperglycemic and insulin culture conditions were visualized with H&E (Figures 4A,D). Higher intensity Picrosirius Red staining was observed in insulin treated groups (Figures 4F,H **)** compared to control EG and HG explants (Figures 4E,G). Slightly deeper staining of Safranin O was also evident in both EGI and HGI groups (Figures 4J,L), with lower intensity staining across non-insulin treated synovium (Figures 4I,K). GLUT1 staining was more pronounced in both HG and HGI treated groups (Figures 4O,P) with minimal staining in EG treated specimens (Figures 4M,N).

### Study 2: Markers of Compromised Glucose Transport and Insulin Resistance in Healthy and OA Synovium Exposed to Hyperglycemic Culture Conditions and Insulin Treatment

**FIGURE 5:**
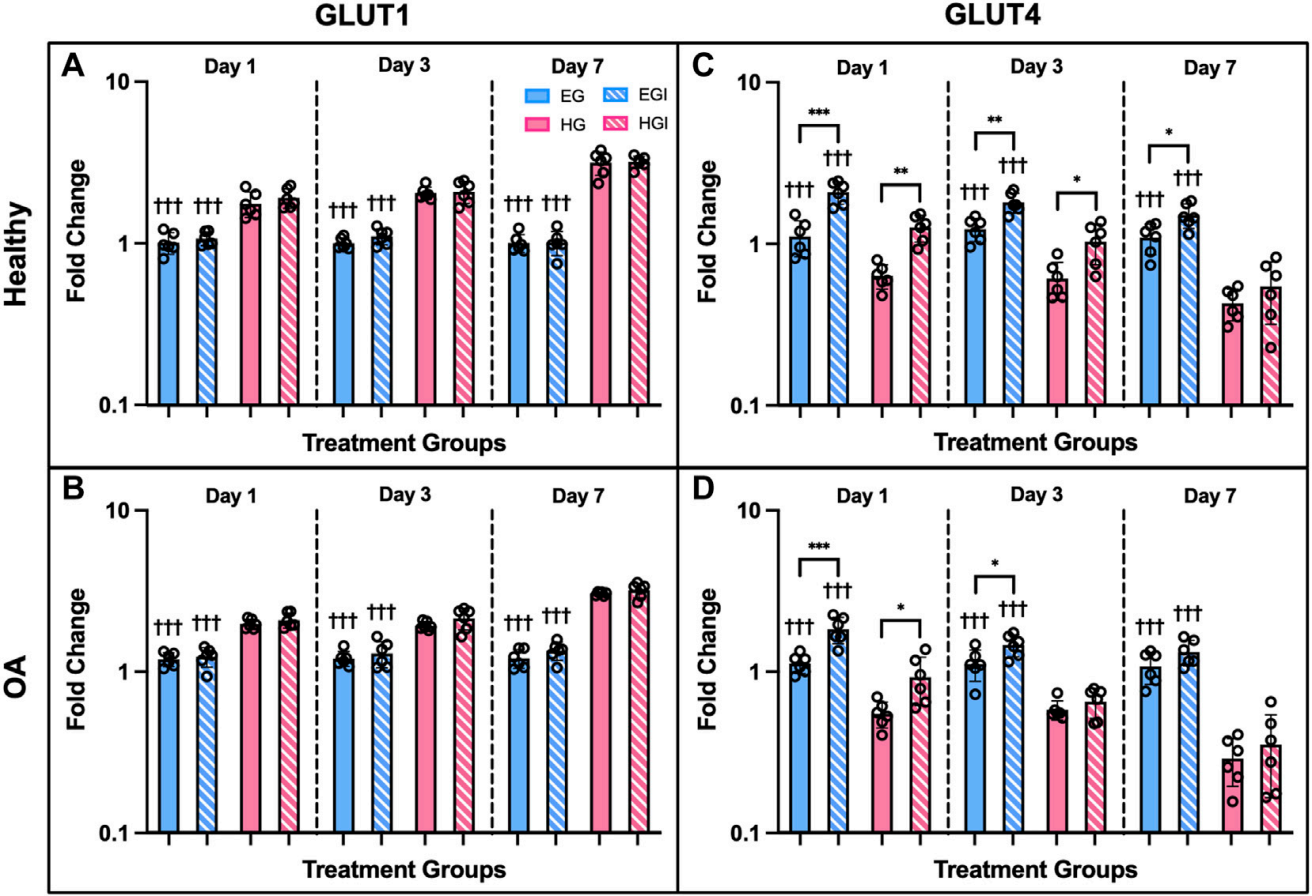
Gene expression of healthy and OA FLS 1, 3 and 7 days after EG, EGI, HG, HGI treatment (*n* = 6), **(A,B)** GLUT1, **(C,D)** GLUT4. †*p* < .05, ††*p* < .01, †††*p* < .0001 (EG vs. HG). *p < .05, **p < .01, ***p < .0001 (control vs. insulin).

Gene expression of hyperglycemic and insulin treated FLS showed significant differences between culture conditions, phenotype, and treatment timepoints. GLUT1 was upregulated under HG conditions compared to EG across all three timepoints and both phenotypes (Figures 5A,B; *p* < .0001). No significant differences in GLUT1 expression were observed between insulin and control groups. For healthy and OA FLS, GLUT4 was downregulated under HG conditions compared to EG across all timepoints (Figures 5C,D; *p* < .0001). Insulin treated groups exhibited elevated GLUT4 expression compared to control across most timepoints. At day 1, GLUT4 expression was significantly upregulated for EGI treated FLS compared to EG for both phenotypes (*p* < .0001), but this difference was less profound between HG and HGI groups (*p* = .0002 for healthy and *p* = .0171 for OA). At day 3, GLUT4 remained elevated for EGI groups compared to control for both healthy and OA cells (*p* = .0007 and *p* = .0311, respectively). Insulin treatment increased GLUT4 expression for HG treated healthy FLS (*p* = .0170), but no significant changes were observed in the OA phenotype. By day 7, GLUT4 levels were attenuated for all insulin treated groups with a significant difference only observed in EGI treated healthy FLS compared to control (*p* = .0160).

**FIGURE 6:**
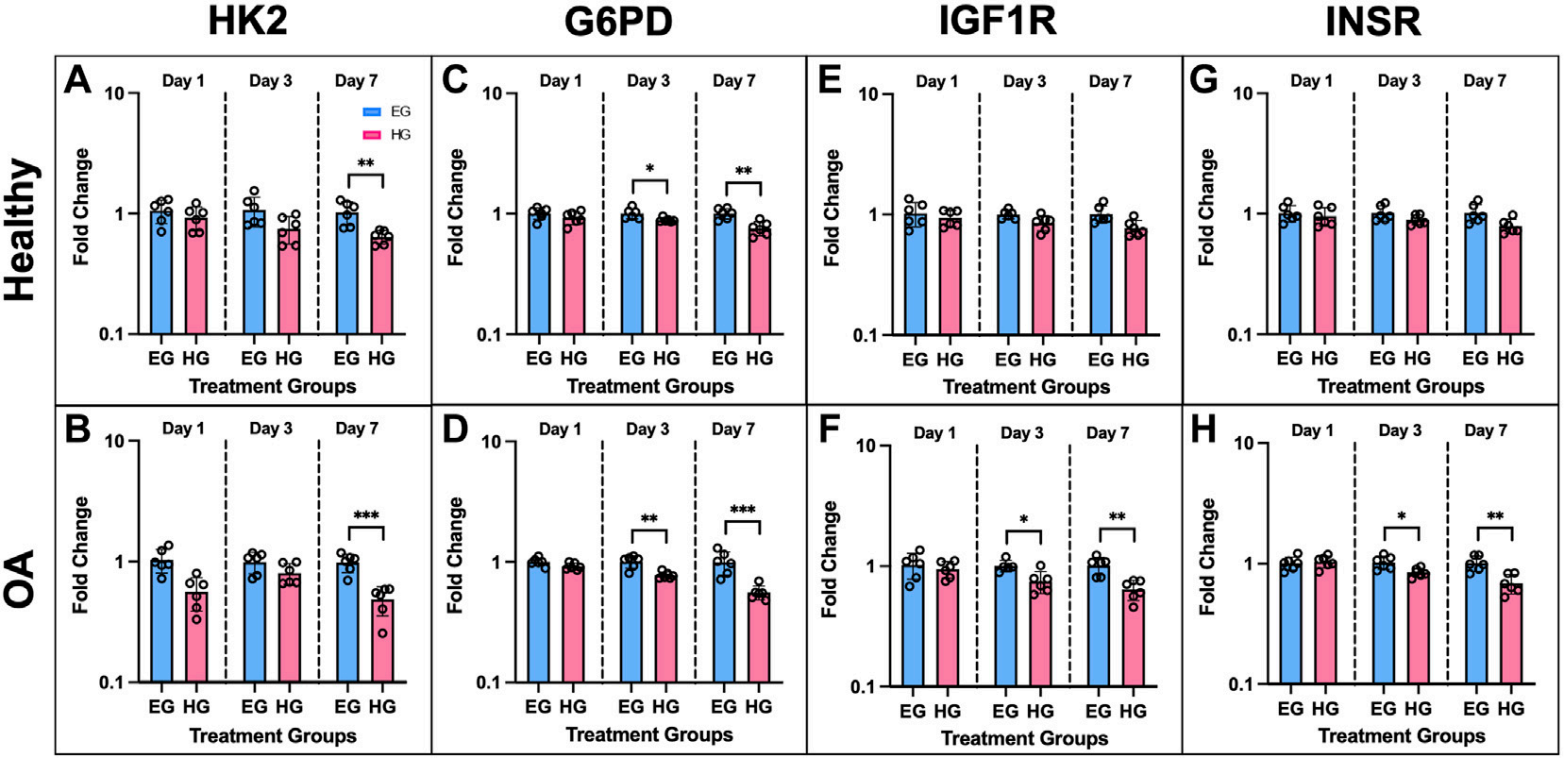
Gene expression of healthy and OA FLS 1, 3 and 7 days after EG and HG treatment (*n* = 6), **(A,B)** HK2, **(C,D)** G6PD, **(E,F)** IGF1R, **(G,H)** INSR. *p < .05, **p < .01, ***p < .0001 (EG vs. HG).

Expression of additional genes involved in cellular glucose regulation *via* glycolysis also yielded significant differences between EG and HG culture conditions. The following genes were mostly downregulated in FLS HG culture: HK2, G6PD, IGF1R, and INSR (Figures 6A–H). HK2 expression was significantly downregulated on day 7 for both phenotypes under HG culture media (Figures 6A,B; *p* = .0030 for healthy and *p* < .0001 for OA). G6PD expression remained consistent at day 1, but was significantly downregulated in HG treated FLS by day 3 (Figures 6C,D; *p* = .0199 for healthy and *p* = .0012 for OA). Day 7 G6PD expression showed more profound decreases in HG groups compared to EG for both healthy and OA cells (*p* = .0017 and *p* < .0001 respectively). IGF1R expression was downregulated only in HG treated OA FLS at days 3 and 7 (Figure 6F; *p* = .0122 and *p* = .0014). Across all timepoints, no significant changes in IGF1R expression between EG and HG groups were observed in healthy FLS (Figure 6E). INSR expression in OA FLS was also downregulated for HG samples compared to EG at day 3 and 7 (Figure 6H; *p* = .0139 and *p* = .0020, respectively), while no significant changes in INSR levels were observed in the healthy group (Figure 6G).

**FIGURE 7:**
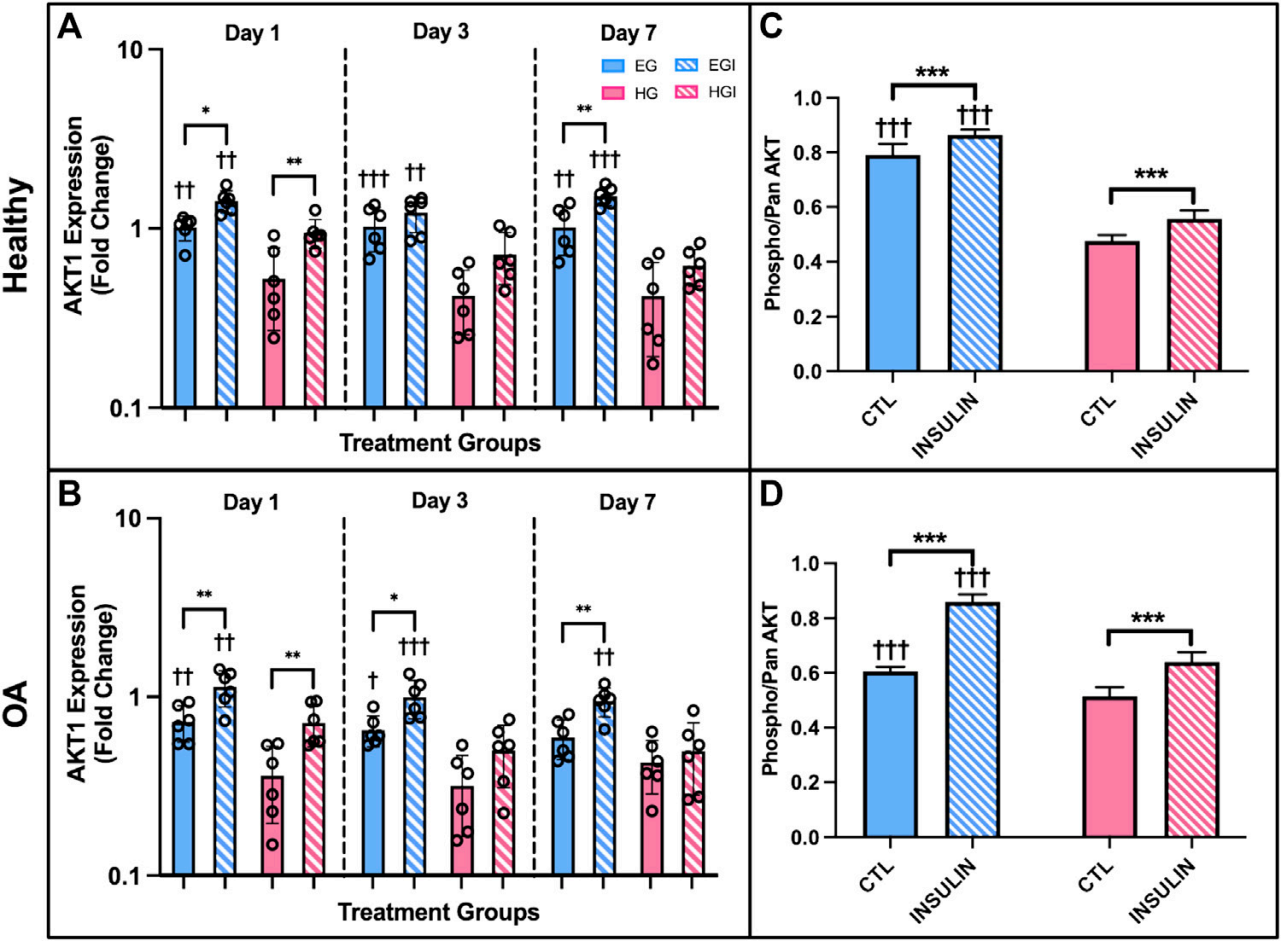
**(A,B)** PCR results of AKT1 gene expression of healthy and OA FLS 1, 3 and 7 days after EG, HG, and insulin treatment. **(C,D)** ELISA results of relative phospho-AKT levels of healthy and OA synovial explants in response to hyperglycemic culture conditions and insulin treatment. †*p* < .05, ††*p* < .01, †††*p* < .0001 (EG vs. HG). *p < .05, **p < .01, ***p < .0001 (control vs. insulin).

AKT1 expression of healthy and OA FLS was significantly downregulated under HG culture conditions. In healthy cells, HG groups exhibited attenuated AKT1 levels compared to EG across all three timepoints (Figure 7A, *p* = .002 for day 1, *p* < .0001 for day 3, *p* = .003 for day 7). AKT1 expression was lower in HG treated OA FLS with significant differences observed of days 1 and 3 (Figure 7B, *p* = .0067 and *p* = .0141, respectively). Insulin exposure mostly increased AKT1 levels compared to control EG and HG groups for both phenotypes. In OA FLS, AKT1 expression in EGI groups was significantly higher than EG across all timepoints (*p* = .0016 for day 1, *p* = .0115 for day 3, *p* = .0086 for day 7). AKT1 levels were also elevated in EGI groups of healthy cells at days 1 and 7 (*p* = .0111 and *p* = .0013, respectively). HGI treated FLS only exhibited increased AKT1 expression compared to control HG groups on day 1 for both healthy and OA cells (*p* = .0092 and *p* = .0085, respectively), while no significant changes were observed at later timepoints.

AKT levels were also confirmed using healthy and OA explants cultured in hyperglycemic and insulin treated media with an ELISA. Relative phospho-AKT was significantly elevated in EG compared to HG groups (*p* < .0001) for both healthy and OA explants (Figures 7C,D) after 7 days of glycemic and insulin treatment. Comparing between control EG groups across phenotypes, phospho-AKT levels were elevated in the healthy explants and slightly attenuated in OA specimens (∼19% decrease). Insulin treatment significantly increased levels of AKT phosphorylation compared to the respective non-insulin treated groups across both EG and HG conditions for OA synovium (*p* < .0001). In the healthy explants, relative phospho-AKT was also elevated for EGI and HGI groups compared to controls (*p* < .0001 and *p* = .0005, respectively).

### Study 3: Intracellular Calcium Response ([Ca^2+^]_i_) to Fluid-Induced Shear Stress and Primary Cilia Properties of Healthy and Non-Diabetic OA FLS Cultured in Hyperglycemic and Insulin Media

**FIGURE 8:**
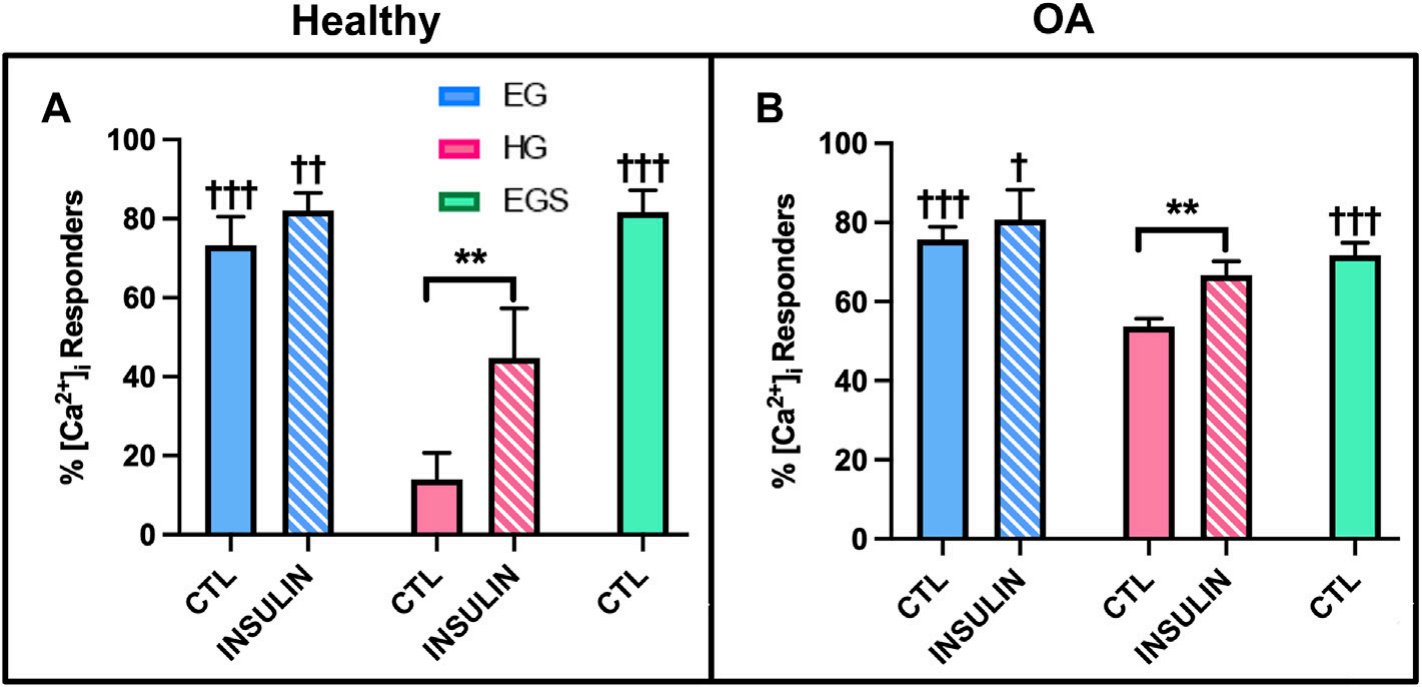
Shear-induced [Ca^2+^]_i_ response with glucose, sucrose, and insulin treatment for **(A)** healthy and **(B)** non-diabetic OA FLS. †*p* < .05, ††*p* < .01, †††*p* < .0001 (EG vs. EGS vs. HG and EGI vs. HGI). *p < .05, **p < .01, ***p < .0001 (control vs. insulin).

FLS demonstrated a robust calcium signaling response to fluid shear (Hamada et al., 2016). The [Ca^2+^]_i_ response for healthy FLS was significantly elevated in the control EG group compared to HG treated cells (73.3 ± 7.3% responders vs. 14.0 ± 6.7%, *p* < .0001, Figure 8A). While the OA FLS response to shear was elevated in the EG treated cells compared to HG (*p* < .0001), the hyperglycemic group had a greater response to shear with 53.6 ± 4.1% responders compared to healthy FLS (Figure 8B). The percent response of the osmotic control consisting of EG media supplemented with sucrose (EGS) yielded similar results to EG treatment across both phenotypes, confirming no osmolarity effects. FLS response to insulin treatment was significantly greater in the EGI compared to the HGI groups for both the healthy and diseased cells (*p* = .0002 and *p* = .0103, respectively). Compared to non-insulin treated FLS, insulin exposure significantly increased percentile response for HGI treated groups for healthy and OA cells (*p* = .0007 and *p* = .0073, respectively). Specifically, the HGI group for the healthy FLS exhibited a significant increase in response that yielded similar results to those of native OA cells, with 44.7 ± 12.7% responders for the heathy HGI group vs. 53.6 ± 4.1% for the OA HG group.

**FIGURE 9:**
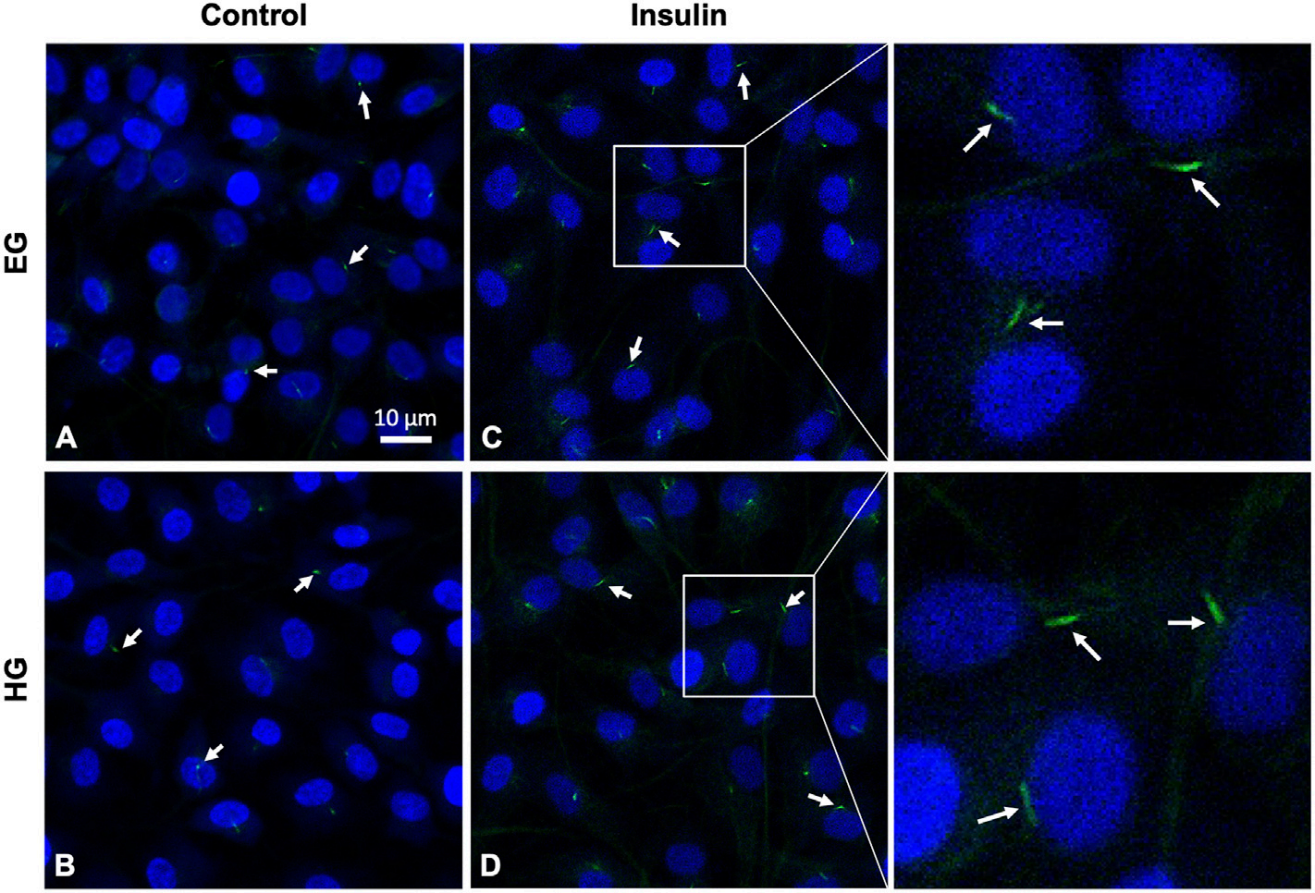
Representative healthy FLS primary cilia (arrows), with DAPI nuclear counterstaining for visualization under **(A,C)** euglycemic and **(B,D)** hyperglycemic culture conditions ± insulin exposure.

**FIGURE 10:**
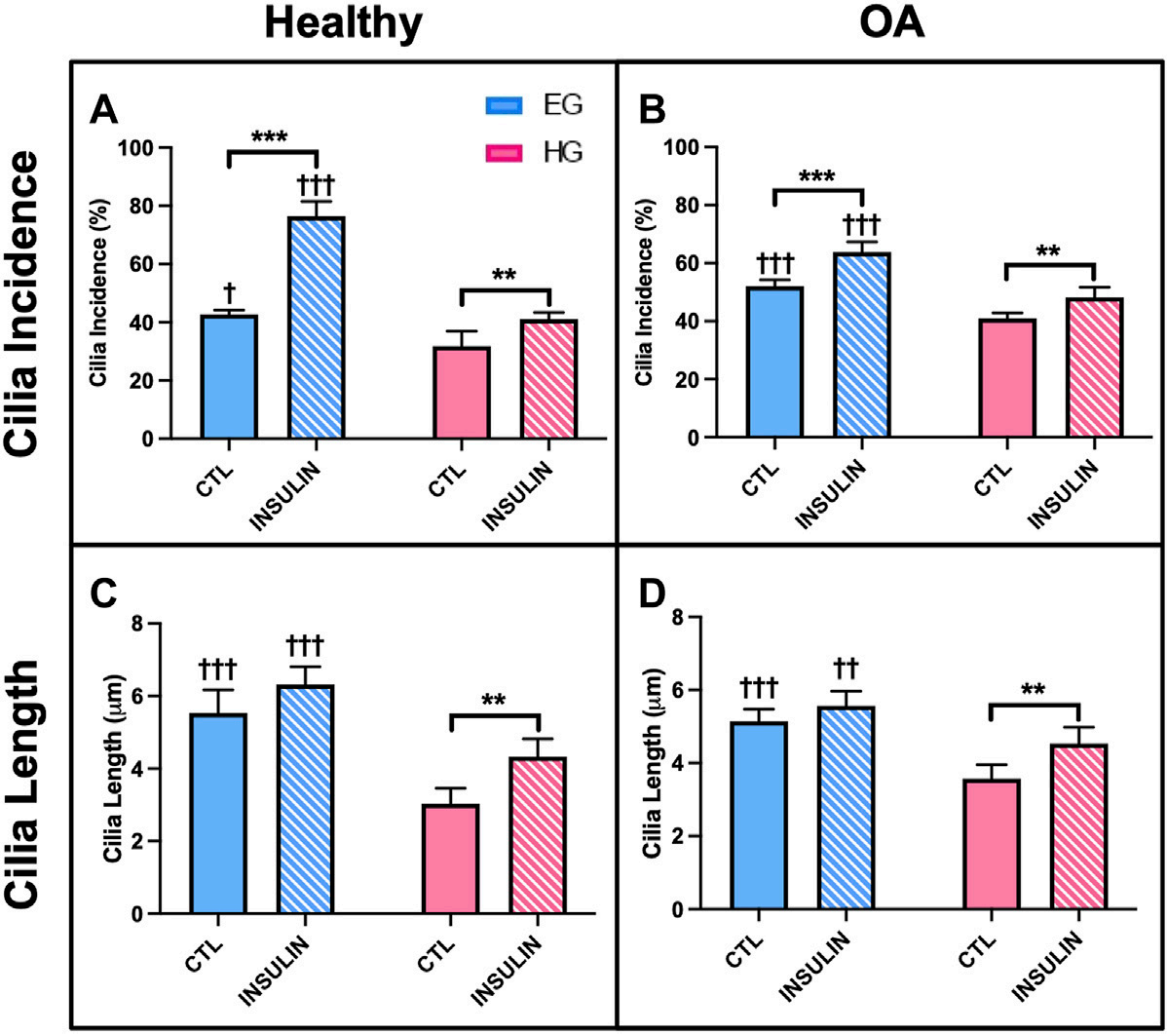
Healthy and non-diabetic OA FLS **(A,B)** incidence and **(C,D)** length of primary cilia detected under glucose and insulin culture conditions. Cilia measurements were pooled across triplicate slides per group †*p* < .05, ††*p* < .01, †††*p* < .0001 (EG vs. HG). *p < .05, **p < .01, ***p < .0001 (control vs. insulin).

HG treatment led to decreased incidence of primary cilia compared to EG groups across both phenotypes (Figures 9A,B, 10A,B; *p* = .0210 for healthy and *p* < .0001 for OA). Insulin treatment significantly increased cilia incidence in EG treated cells for both healthy and diseased FLS (Figures 9C, 10A,B; *p* < .0001). HGI groups also exhibited increased cilia incidence compared to HG in both healthy and OA cells (Figures 9D, 10A,B; *p* = .0084 and *p* = .0052, respectively), but this difference compared to control HG groups was not as profound as EG and EGI treatment. In addition, HG treatment significantly decreased average cilia length for both phenotypes (Figures 10C,D; *p* < .0001). Insulin treatment significantly increased cilia length for HGI groups compared to control HG groups for both the healthy and OA FLS (*p* = .0053 and *p* = .0062, respectively).

### Study 4: AKT Phosphorylation and Upstream Markers of Cellular Activity Under Hyperglycemic and Insulin Culture Conditions for Diabetic OA Synovium

**FIGURE 11:**
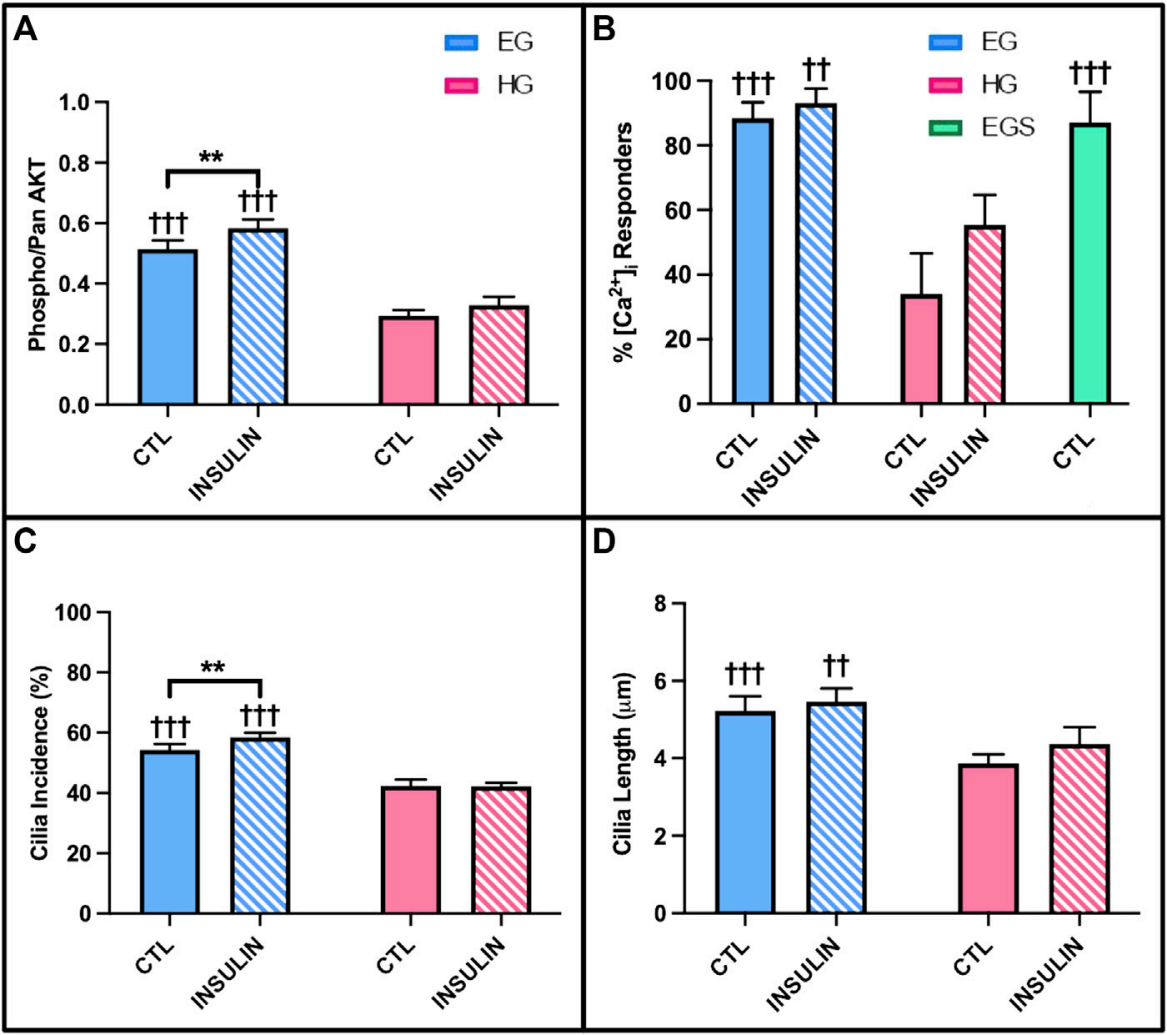
Diabetic OA synovial explants **(A)** relative phospho- AKT levels in response to hyperglycemic culture conditions and insulin treatment. Diabetic OA FLS **(B)** shear-induced [Ca^2+^]_i_ response with glucose, sucrose, and insulin exposure and **(C)** incidence and **(D)** length of primary cilia. †*p* < .05, ††*p* < .01, †††*p* < .0001 (EG vs. HG). *p < .05, **p < .01, ***p < .0001 (control vs. insulin).

AKT phosphorylation levels, calcium response to shear stress, and cilia properties were investigated for diabetic OA synovium under the same glucose and insulin treatment conditions as the previous studies. Similar to the healthy and non-diabetic OA samples, relative phospho-AKT was significantly reduced in HG treatment compared to control EG groups (*p* < .0001) for the diabetic OA explants (Figure 11A)**.** Comparing EG and HG groups across all three phenotypes, the diabetic OA explants exhibited the greatest decrease in phospho-AKT levels between both glycemic conditions (∼43% decrease between control EG and HG groups). While insulin exposure increased AKT phosphorylation in EGI groups compared to the control EG treatment (*p* = .0040) in the diabetic phenotype, no significant changes in AKT phosphorylation were observed between the insulin and non-insulin treated HG groups.

The [Ca^2+^]_i_ response to fluid shear of diabetic OA FLS was significantly elevated under EG culture conditions compared to HG treatment with 88.3 ± 4.9% vs. 34.0 ± 12.5% responders, respectively (Figure 11B; *p* < .0001). Insulin treated HGI groups also exhibited decreased [Ca^2+^]_i_ response to shear compared to EGI (*p* = .0004). The response of EGS and EG groups suggest no osmolarity effects. While insulin increased FLS response under HG culture conditions in the healthy and non-diabetic OA FLS (Figures 8A,B), no significant changes were observed between insulin treated cells and non-insulin groups in the diabetic OA phenotype (*p* = .9961).

Hyperglycemic and insulin treatment of diabetic OA FLS yielded similar trends in cilia incidence and length compared to healthy and OA cells from study 3. Across both glycemic culture conditions, HG treatment decreased primary cilia incidence compared to EG groups for the diabetic OA FLS (Figure 11C; *p* < .0001). Insulin exposure in the HGI groups also exhibited attenuated cilia incidence compared to EGI treated cells (*p* < .0001). However, insulin treatment did increase cilia incidence in the EGI groups compared to control EG (*p* = .0083). Unlike the healthy and OA FLS (Figures 10A,B), no significant changes in cilia incidence were observed between insulin and non-insulin treated HG groups (*p* = .9980) for the diabetic OA phenotype. For cilia length measurements, HG treatment significantly decreased cilia length in the diabetic OA cells (Figure 11D; *p* < .0001). While insulin increased average cilia length for HGI groups compared to HG groups in both healthy and non-diabetic OA cells (Figures 10C,D), no significant changes in cilia length were observed between the insulin and non-insulin treated EG and HG groups for the diabetic OA FLS.

## Discussion

Synovial fibroblasts are significant contributors to the articular cartilage environment by producing molecules important for lubrication, ECM remodeling, and regulating solute transport in and out of the joint (Hung et al., 1995; Denko et al., 1996; Harsha and Joyce, 2011; Hoey et al., 2012; Stefani, 2020). However, synovium function is compromised in patients with DM (Griffin and Huffman, 2016). It is known that the development of DM promotes synovial inflammation and insulin resistance in progression of the OA disease state (Griffin and Huffman, 2016; Li et al., 2021). In FLS isolated from non-diabetic patients with OA, it has been shown that insulin inhibits the production of inflammatory cytokines (de Luca and Olefsky, 2009; Griffin and Huffman, 2016). However, in diabetic OA patients, synovial insulin signaling is further compromised, indicative that the anti-inflammatory and anti-catabolic function of insulin is suppressed in the presence of both comorbidities (Griffin and Huffman, 2016; Veronese et al., 2019; Tchetina et al., 2020). In the current study, we sought to further the understanding of this correlation between hyperglycemia and insulin resistance that results from the coexistence of both OA and DM.

In healthy specimens, HG treatment decreased DNA levels compared to EG groups (Figure 3A), potentially indicative of greater cell damage, characteristic of the diabetic disease state (Brownlee, 2005). Collagen and GAG content of both healthy and OA explants were also affected by HG treatment and insulin exposure (Figures 3C–F), suggesting glycemic environments may affect cell sensitivity and metabolism (Hayden et al., 2005; Nagy et al., 2019). While lower levels of GAG and collagen have been shown to be characteristic features of the diabetic disease state, insulin treatment recovered these matrix constituents across all HG groups for both healthy and OA explants, indicative of its pro-anabolic effect (Stultz and Edelman, 2003; Gowd et al., 2016). Media analysis also confirmed that increased GAG in HG treated tissue contributed to ECM remodeling across both phenotypes (Figures 3G,H). Furthermore, HG treatment has also been associated with lower NO release in media under insulin resistant conditions, characteristic of DM (Figures 3I,J) (Hoshiyama et al., 2004). Insulin treatment did not have a significant effect on both media GAG and NO release, suggesting the inability of insulin to restore the effect of HG culture conditions to baseline levels exhibited under EG treatment. Histological analysis confirmed the differences observed in GAG and collagen levels under HG media conditions. The high intensity staining of GLUT1 was also observed in the HG groups (Figures 4O,P), suggesting that high glucose environments may alter glucose regulation and insulin activity in healthy and diseased synovium.

In the current study, reciprocal expression of the glucose transport proteins GLUT1 and GLUT4 was observed under EG and HG culture conditions in both healthy and OA FLS (Ebeling et al., 1998). Under HG culture conditions, GLUT1 expression was upregulated, while GLUT4 levels were significantly attenuated (Figures 5A–D). GLUT1 is expressed ubiquitously and operates independent of insulin activity, while GLUT4 is activated through translocation to cell membranes under insulin dependent mechanisms (Ebeling et al., 1998; Bryant et al., 2002, Gallagher et al., 2020). Insulin exposure had no effect on GLUT1 expression across all timepoints and both media conditions, potentially indicative of the insulin independent effect of hyperglycemia on GLUT1 levels. However, insulin significantly increased GLUT4 expression at day 1, but this difference was reduced across each subsequent timepoint. By day 7, no significant differences were observed in GLUT4 levels between insulin and non-insulin treated HG groups, supporting the reduced protective role of insulin in counteracting the elevated glucose levels and potentially indicative of insulin resistance. Overall, OA FLS exhibited lower GLUT4 levels for HG treated samples compared to healthy cells, suggesting a link between OA and diabetic insulin resistance. The lower GLUT4 expression under HG treatment supports the decreased levels of AKT phosphorylation observed in the HG treated synovial explants. Further in the glycolysis pathway, glucose that enters the cell via GLUT proteins is phosphorylated by hexokinase to G6PD (Ebeling et al., 1998; Tchetina et al., 2020). The expression of both HK2 and G6PD in HG treated FLS were significantly lower compared to control EG across both healthy and OA cells (Figures 6A–D), potentially indicative of the diabetic disease state (Esteves et al., 2018). The expression of IGF1R and INSR was also lower in HG treated FLS (Figures 6E–H), suggesting the diminished effect of insulin in regulating glucose uptake under diabetic and possible insulin resistant conditions.

The AKT phosphorylation cascade is a specific signaling pathway that connects glycolysis markers implicated in cellular glucose uptake and insulin activity (Garcia-Carbonell et al., 2016). AKT is involved in several complex signaling networks that regulate various cellular functions including cell metabolism, proliferation, motility, and apoptosis (Jazirehi et al., 2012; Du et al., 2019). It has also been implicated in numerous comorbidities including diabetes, cardiovascular, and neurodegenerative diseases (Zarneshan et al., 2020). In DM, the AKT signaling pathway is a mediator of insulin activity and plays a crucial role in disease pathogenesis (Hamada et al., 2016; Huang et al., 2018; Gabbouj et al., 2019). It promotes the metabolic effects of insulin and facilitates glucose transport, lipid synthesis, gluconeogenesis, and glycogen synthesis (Boucher et al., 2014; Huang et al., 2018). AKT is specifically involved in insulin signaling as the activation of insulin receptors triggers a phosphorylation cascade, which is initiated by receptor autophosphorylation and the activation of insulin receptor substrate proteins (Boucher et al., 2014). The activation of insulin receptors drives the phosphorylation of AKT, thus contributing to the translocation of GLUT4 to the plasma membrane (Boucher et al., 2014; Beg et al., 2017; Nitulescu et al., 2018). Therefore, AKT is expressed in insulin-responsive tissues, including synovium, and functions to regulate glucose metabolism (Nitulescu et al., 2018). Studies have shown that AKT deletion in knockout mice has been correlated with insulin resistance, hyperinsulinemia, and glucose intolerance (Cho et al., 2001; Nitulescu et al., 2018). In addition, defects in AKT signaling have also been correlated with diabetic insulin resistance in humans (Nitulescu et al., 2018). The results of the current study support the effect of hyperglycemic culture conditions on AKT signaling and phosphorylation in a model of diabetic insulin resistance. Both healthy and diseased synovial explants demonstrated that hyperglycemia led to decreased phospho-AKT levels compared to the respective control EG groups (Figures 7C,D, 11A). PCR analysis yielded consistent results with lower AKT1 expression in HG treated FLS compared to EG across all timepoints and both phenotypes (Figures 7A,B). Previous studies have shown that the attenuation of AKT phosphorylation levels is indicative of more insulin resistant environments (Tonks et al., 2013). Therefore, the greater decrease in AKT phosphorylation under HG culture conditions for the diabetic explants may suggest a more insulin resistant phenotype compared to healthy and non-diabetic OA tissue. For the insulin treated groups, relative phospho-AKT was elevated in the EGI and HGI groups for both healthy and non-diabetic explants. However, the difference in AKT phosphorylation between the insulin and non-insulin treated groups for the diabetic OA synovium did not show significant differences under HG culture conditions, potentially indicative of greater insulin resistance.

Since the AKT signaling pathway and markers of glycolysis have been shown to be controlled by upstream calcium influxes, intracellular calcium transients in response to fluid-induced shear were investigated in study 3 (Nicholson-Fish et al., 2016). Hyperglycemic culture conditions have been correlated with abnormal calcium signal transduction, which may be linked to reduced insulin responsiveness (Boldizsár et al., 2002; Lebeche et al., 2008) We observed that HG treated FLS exhibited fewer percent responders compared to EG groups, potentially indicative that high glucose environments decrease cellular responses and possibly affect cell function. For healthy and non-diabetic OA FLS, insulin treatment increased [Ca^2+^]_i_ response for HGI treated groups (Figures 8A,B), suggesting that insulin recovers the cell response of HG treated FLS to levels closer to the control EG groups. However, insulin exposure did not affect the number of cells that responded to shear stress for the native diabetic OA phenotype (Figure 11B), indicative of lower insulin responsiveness and potentially diabetic insulin resistance.

The incidence and length of primary cilia provides a potential mechanism to support changes in fluid shear-induced [Ca^2+^]_i_ responses among the different glucose and insulin treated FLS groups. Dysfunctional primary cilia due to hyperglycemia have been shown to inhibit mechanotransduction and cellular responses to external stimuli (Estell et al., 2017; Ritter et al., 2018; Kluth et al., 2019; Stefani et al., 2019). In study 3, HG treatment decreased cilia incidence and length across healthy and OA FLS (Figures 10A–D), consistent with the decrease in [Ca^2+^]_i_ response of FLS under hyperglycemic conditions for both phenotypes. However, insulin exposure increased cilia incidence and length for HGI groups compared to control HG treatment for both healthy and non-diabetic OA FLS, further supporting the difference in insulin responders between the two phenotypes. Interestingly, the diabetic OA FLS displayed no significant changes in primary cilia length and incidence between insulin and non-insulin treated HG groups (Figures 11C,D), potentially indicative of decreased insulin responsiveness in recovering the effects of the high glucose environment.

Overall, the current study demonstrated that hyperglycemic treatment and insulin exposure affects synovium ECM properties. Hyperglycemic culture conditions also alter the expression of cell insulin receptors, glucose transporters, and specific glycolysis markers implicated in glucose uptake and breakdown. A correlation appears to exist between relative levels of AKT phosphorylation, percent responders to fluid shear, and cilia incidence and length. Specifically, it is known that OA environments increase synoviocyte sensitivity to fluid shear by altering intercellular communication, which may affect downstream functions such as decreased AKT phosphorylation and altered glycolysis markers, implicated in compromised insulin activity that contribute to progression of the diabetic disease state (Wilcox, 2005; Boucher et al., 2014).

Potential limitations associated with this research include a control, healthy synovium group that was significantly younger and all male, compared to the diseased tissue groups (OA ± DM). The latter reflects the inherent challenges of procuring normal tissues of any age for such studies and higher female incidence of OA. While the current study using healthy tissue exposed to HG conditions revealed that normal synovium is very sensitive to changes in glucose and insulin levels compared to OA-diseased tissue, similar trends across all three phenotypes were observed. The latter is even more remarkable in that the findings were observed using tissue from donor ages spanning 4 decades (19–80 years old) and despite sex-composition differences between the groups. In this context, these findings are supportive of our contention that healthy synovium with a high glucose culture-induced diabetic phenotype can serve as a model of diabetic insulin resistance.

To better understand the connection between OA and DM, future work will continue to explore strategies to induce the diabetic phenotype from healthy tissue. Preconditioning media with various hyperglycemic concentrations will be optimized to confer the diabetic phenotype and overcome confounding factors associated with age or the disease state of patients undergoing total knee arthroplasty. With superposition of pro-inflammatory cytokines (Stefani et al., 2019), this tissue platform would permit simulated study of DM with the comorbidity of OA, as well as the evaluation of therapeutic strategies aimed at their mitigation. Using the *in vitro* diabetic insulin resistance model system reported here, the action of peroxisome proliferators activated receptors (PPAR), involved in regulating joint inflammation and glucose homeostasis, and other biochemical cascades can be investigated in future studies (Li et al., 2017).

## Data Availability

The original contributions presented in the study are included in the article/Supplementary Files, further inquiries can be directed to the corresponding author.
